# The ATPase activity of the phosphatidylethanolamine flippase TAT-5 inhibits extracellular vesicle budding from the plasma membrane

**DOI:** 10.17912/micropub.biology.000779

**Published:** 2023-03-24

**Authors:** Lauren R Pitts, Julia Frondoni, Alexander T Nguyen, Ann M Wehman

**Affiliations:** 1 Biological Sciences, University of Denver, Denver, Colorado, United States

## Abstract

Cells release extracellular vesicles (EVs) from their surface, but the mechanisms that govern EV release by plasma membrane budding are poorly understood. The lipid flippase TAT-5 inhibits EV release from the plasma membrane in *C. elegans* , but how the level of flippase activity regulates EV release was unknown. We generated point mutations in the DGET motif of TAT-5 predicted to lead to a partial or complete loss of ATPase activity. We discovered that *tat-5(E246Q)* mutants were sterile, while *tat-5(D244T)* mutants produced embryos that arrested during development. Using degron-based reporters, we found that EV release was increased in *tat-5(D244T)* mutant embryos and that phagocytosis was also disrupted. These data suggest that a low level of flippase activity can promote fertility, while a higher level of flippase activity is required to inhibit EV release, allow phagocytosis, and carry out embryonic development.

**Figure 1. Mutations in the DGET motif of TAT-5 alter fertility, phagocytosis, and extracellular vesicle (EV) release:**
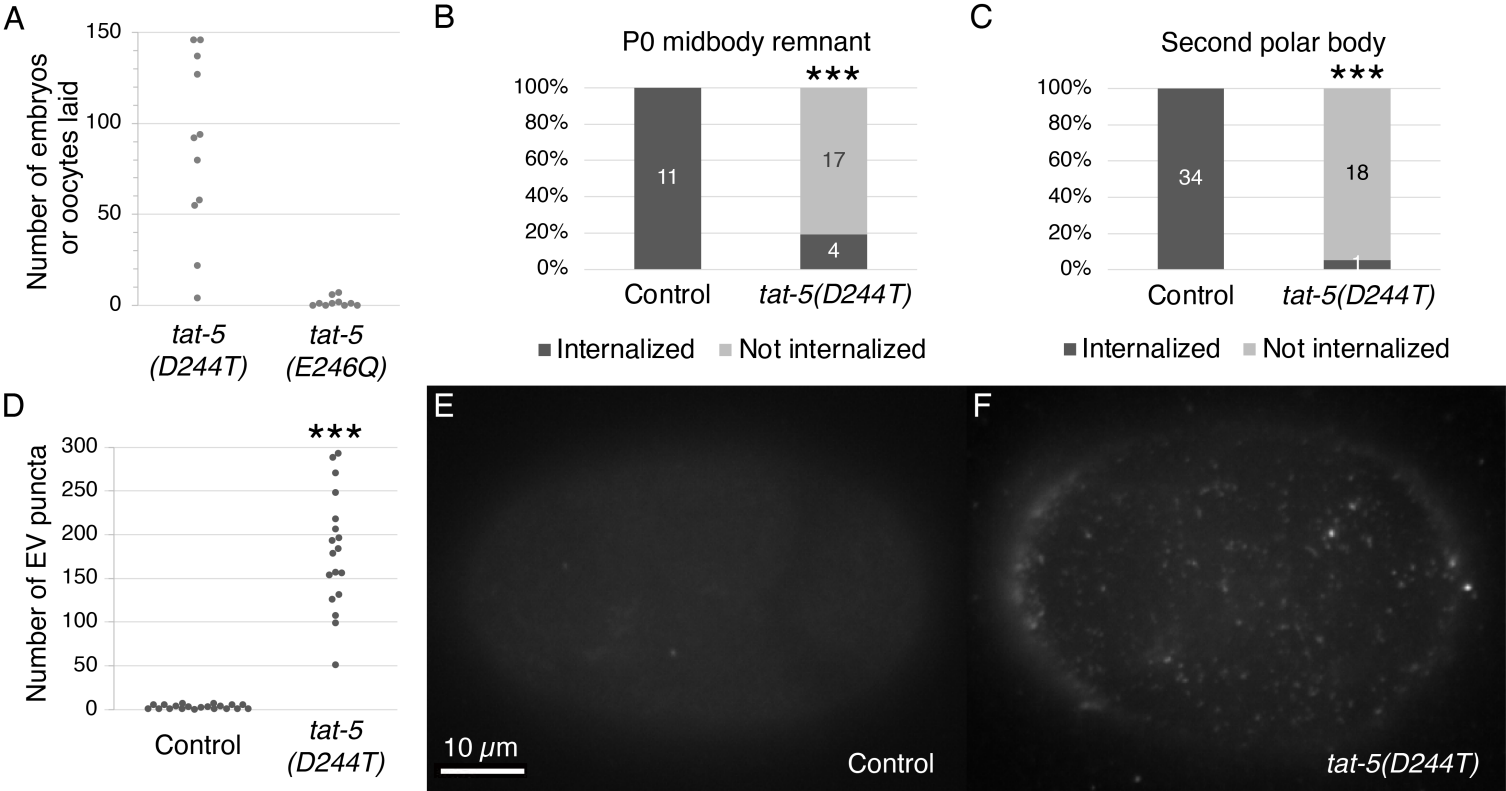
(A) Embryos and oocytes were laid by *tat-5(D244T)* mutant hermaphrodites (n=11), but rarely by *tat-5(E246Q)* mutant hermaphrodites (n=10). (B-C) The phagocytosis of P0 midbody remnants labeled with NMY-2::mCh (B) or second polar bodies labeled with mCh::PH::CTPD (C) was significantly disrupted in *tat-5(D244T)* mutant embryos (***p<0.001 Fisher’s exact test). (D) The number of mCh::PH::CTPD puncta on the surface of individual *tat-5(D244T)* mutant embryos (n=18) was significantly increased compared to control embryos (n=20, ***p<0.001 Student’s t-test). (E-F) Surface of 4-cell embryos expressing mCh::PH::CTPD in control (E) and *tat-5(D244T)* mutants (F).

## Description

The lipid flippase TAT-5 inhibits extracellular vesicle (EV) release from the plasma membrane and maintains phosphatidylethanolamine asymmetry in *C. elegans* (Wehman et al., 2011), but it is not understood how lipid asymmetry regulates plasma membrane budding to release EVs. EV release is also inhibited by a homolog of TAT-5, ATP9A, in human cells (Naik et al., 2019), suggesting that these homologs have a conserved role in EV regulation. TAT-5 and ATP9A are P4B-ATPases like yeast Neo1, which has been shown to hydrolyze ATP to flip lipids in membranes (Bai et al., 2021). Therefore, it is important to determine how modulating P4B-ATPase flippase activity influences EV release. Previous studies on the mammalian P4A-ATPase ATP8A2 have shown that mutations in the DGET motif found in the cytosolic Actuator domain of P4-ATPases can lead to graded effects on ATPase activity and lipid transport. A D to T mutant (D196T) resulted in a 3-fold loss in ATP hydrolysis and lipid flipping, while an E to Q mutant (E198Q) resulted in a complete loss of ATP hydrolysis and lipid flipping (Coleman et al., 2012). Thus, the DGET motif is a promising target to alter TAT-5 flippase activity and determine its effect on EV production.

We previously found that expression of a GFP::TAT-5(E246Q) transgene failed to suppress EV release or rescue the sterility and maternal-effect embryonic lethality of *tat-5* deletion mutants (Wehman et al., 2011), suggesting that E246 of the DGET motif is similarly essential for TAT-5 ATPase and flippase activity. However, this transgene was expressed in the germ line and *tat-5* is normally ubiquitously expressed (Lyssenko et al., 2008). Therefore, the transgene could have failed to rescue sterility due to the lack of expression in somatic tissues. To confirm that the DGET motif is essential for TAT-5 function, we used CRISPR/Cas9-mediated genome editing to mutate glutamic acid 246 to glutamine in TAT-5. We found that *tat-5(E246Q)* mutants were mostly sterile, with germ lines laying an average of 1±2 oocytes and 0.5±1 embryos (Fig. 1A), which died during embryogenesis. These phenotypes are similar to *tat-5* deletion mutants (Wehman et al., 2011), suggesting that TAT-5(E246Q) is inactive like ATP8A2(E198Q) (Coleman et al., 2012).

To test the effect of a partial loss of TAT-5 activity on sterility and viability, we used CRISPR/Cas9-mediated genome editing to mutate aspartic acid 244 to threonine in the DGET motif. We found that *tat-5(D244T)* mutants were fertile, laying an average of 77±43 embryos and 11±8 oocytes (Fig. 1A). However, the embryos rarely hatched, similar to RNAi knockdown of *tat-5* causing embryonic lethality (Wehman et al., 2011). These data suggest that *tat-5(D244T)* is a partial loss-of-function allele, similar to the partial decrease in lipid transport and ATPase activity observed with ATP8A2(D196T) (Coleman et al., 2012), and raise the possibility that low levels of TAT-5 ATPase activity are sufficient for fertility in hermaphrodites, but higher levels are needed for embryonic development.

As increased EV release is correlated with gastrulation defects and embryonic lethality (Wehman et al., 2011, Beer et al., 2018), we next examined the effect of a partial loss of TAT-5 activity on EV production. We used a degron-tagged reporter, mCh::PH::CTPD, which initially labels the plasma membrane by the PH domain binding the lipid PI _4,5_ P _2_ . The C-terminal phosphodegrons (CTPD) cause the intracellular pool of mCh::PH::CTPD to be degraded after the first embryonic cell division, leaving EVs already released from the plasma membrane labeled (Beer et al., 2019). We found that control embryos had an average of 3±2 mCh::PH::CTPD puncta on their surface (Fig. 1D-E). In contrast, *tat-5(D244T)* mutants had an average of 181±67 mCh::PH::CTPD puncta (Figure 1D, F), suggesting a >60-fold increase in EV release. These data indicate that despite being able to support fertility, TAT-5(D244T) loses its ability to regulate EV release. This suggests that higher levels of TAT-5 ATPase activity are required to inhibit EV budding from the plasma membrane.

As large increases in EV release are also correlated with defects in phagocytosis (Fazeli et al., 2020), we next investigated polar body and midbody remnant uptake (Fazeli et al., 2016, Fazeli et al., 2018). Using the non-muscle myosin NMY-2::mCh reporter to label midbody remnants (Fazeli et al., 2016), we found that *tat-5(D244T)* mutant embryos were defective in P0 midbody remnant internalization (Figure 1B), which normally occurs by the 6-cell stage (Fazeli et al., 2016). Using the mCh::PH::CTPD reporter to label polar bodies (Beer et al., 2019), we found that *tat-5(D244T)* mutant embryos were defective in second polar body internalization (Figure 1C), which normally occurs by the early 4-cell stage (Fazeli et al., 2018). These results suggest that *tat-5(D244T)* is a strong loss-of-function allele and provide further evidence correlating the overproduction of EVs with the disruption of phagocytic events during embryonic development (Fazeli et al., 2020).

In summary, our studies targeting the DGET motif suggest that robust TAT-5 flippase activity is required to inhibit EV release and allow phagocytosis and embryogenesis, while low levels of TAT-5 flippase activity are sufficient for fertility. While hypomorphic ATPase *tat-5(D244T)* mutants have severe loss-of-function phenotypes, including embryonic lethality, strongly increased EV release, and phagocytic defects, they lay more embryos than *tat-5(E246Q)* mutants, suggesting that D244T does not completely disrupt TAT-5 activity as E246Q does. The *tat-5(D244T)* mutant is therefore a useful resource to determine how TAT-5 ATPase activity regulates lipid asymmetry to alter the levels of EV release from the plasma membrane. As mutations in the TAT-5 homolog ATP9A have recently been shown to cause neural, muscular, fertility, and growth defects in humans and mice (Vogt et al., 2022, Mattioli et al., 2021, Meng et al., 2023), it will be important to tease apart which defects result from EV regulation or the intracellular trafficking roles of ATP9A (Tanaka et al., 2016, McGough et al., 2018). Determining whether novel patient alleles alter ATPase activity and lipid asymmetry could also provide insight into the etiology of disease and potential therapeutic strategies.

## Methods

**Worm strains and maintenance**

*C. elegans* strains were maintained and crossed on Nematode Growth Media (NGM) seeded with OP50 bacteria and grown at room temperature using standard protocols (Brenner 1974). Homozygous mutant worms were selected by the absence of Venus fluorescence in their pharynx. See Table 1 for a list of strains used in this study.

**Table 1: Worm strains**

**Table:** 

Strain	Genotype	Source
N2	Wild Type	Brenner, 1974
PHX2519	*tat-5(syb2519[E246Q]) / tmC18[dpy-5(tmIs1200[myo-2p::Venus])] I*	SunyBiotech
PHX2596	*tat-5(syb2414[D244T]) / tmC18[dpy-5(tmIs1200[myo-2p::Venus])] I*	SunyBiotech
WEH430	*unc-119(ed3) xnIs8[pJN343: nmy-2::NMY-2::mCherry; unc-119(+)] III; xnIs65[nmy-2::gfp::zf1, unc-119(+)] IV*	Beer et al., 2019
WEH434	*unc-119(ed3) III; wurIs155[pAZ132-coPH-oma-1(219-378): pie-1::mCh::coPH::CTPD; unc-119(+)]*	Beer et al., 2019
WEH595	*tat-5(syb2414[D244T]) / tmC18[dpy-5(tmIs1200[myo-2p::Venus])] I; unc-119(ed3) xnIs8[pJN343: nmy-2::NMY-2::mCherry; unc-119(+)] III*	Crossed N2 x WEH430 x PHX2596
WEH599	*tat-5(syb2414[D244T]) / tmC18[dpy-5(tmIs1200[myo-2p::Venus])] I; wurIs155[pAZ132-coPH-oma-1(219-378): pie-1::mCh::coPH::CTPD; unc-119(+)]*	Crossed WEH434 x PHX2596

**Genome editing**

DGET mutant alleles (Table 2) were created by SunyBiotech using CRISPR-Cas9-mediated genome editing (Paix et al. 2014) and verified by sequencing.

**Table 2: DGET mutant *tat-5* alleles.** Mutations are underlined and in bold type.

**Table:** 

**Allele**	**Sequence**
Wild Type	CAATTGGATGGAGAAACTGAT
*syb2414[D244T]*	CAATTG **ACC** GG **T** GAAACTGAT
*syb2519[E246Q]*	CAATTGGATGG **CCAA** ACTGAT

**Genotyping worms by PCR**

Worms were lysed and *tat-5* DNA was amplified using 2X OneTaq polymerase (New England BioLabs) and the primers listed in Table 3. The *tat-5(D244T)* mutation was detected using an AgeI digest (New England BioLabs) and the *tat-5(E246Q)* mutation can be detected using an MscI digest.

**Table 3: Primers used to genotype worm strains.**

**Table:** 

**Primer**	**Sequence**
tat-5 geno F	TGC TCC AAT CAC TTA CTG GGG AC
tat-5 exon 5 R KpnI	CCG GTA CCT TTC ATG GCA ACC ATA ACC

**Embryo and oocyte counts**

L4 hermaphrodite larvae were singled onto 24-well plates. Embryos and oocytes laid on the plate were counted on an Olympus SZ61 one day later.

**Light microscopy**

Fluorescence image stacks were collected with a Zeiss Axio Observer 7 inverted microscope with a Plan-Apo 40X 1.4 NA oil objective with Excelitas Technologies X-Cite 120LED Boost illumination, and a HamamatsuORCA-Fusion sCMOS camera controlled by 3i SlideBook6 software. Control NMY-2::mCh data was collected for Fazeli et al., 2020.

**Image manipulation**

The surface z-section was rotated, cropped, and the brightness was adjusted using Adobe Photoshop 2023.

**Extracellular vesicle counts**

mCh::PH::CTPD puncta were marked and counted on the top surface of 3- to 15-cell embryos using the ImageJ Cell Counter function (FIJI 2.3.0). The number of fluorescent puncta in clusters were estimated according to the average size of discrete mCh::PH::CTPD puncta.

**Midbody remnant or second polar body internalization**

Still images were scored using 3i SlideBook6 software. Internalization was defined as when the cargo was fully inside a cell. Polar bodies were labeled with mCh::PH::CTPD and scored from the 4-cell to 28-cell stage, while midbody remnants were labeled with NMY-2::mCh and scored from the 7- to 15-cell stage. Cell stages were identified using DIC.

**Statistics**

Statistical significance was determined using Student’s one-tailed t-test.
